# Supplementary material for: NT-proBNP Levels Influence the Prognostic Value of Mineral Metabolism Biomarkers in Coronary Artery Disease
Source: J Clin Med. 2022 Jul 17;11(14):4153. doi: 10.3390/jcm11144153 (PMC9319637; doi:10.3390/jcm11144153)
Supplement: Supplementary file 1 [file jcm-11-04153-s001.zip › jcm-1787416-supplementary.pdf]

Supplementary Material

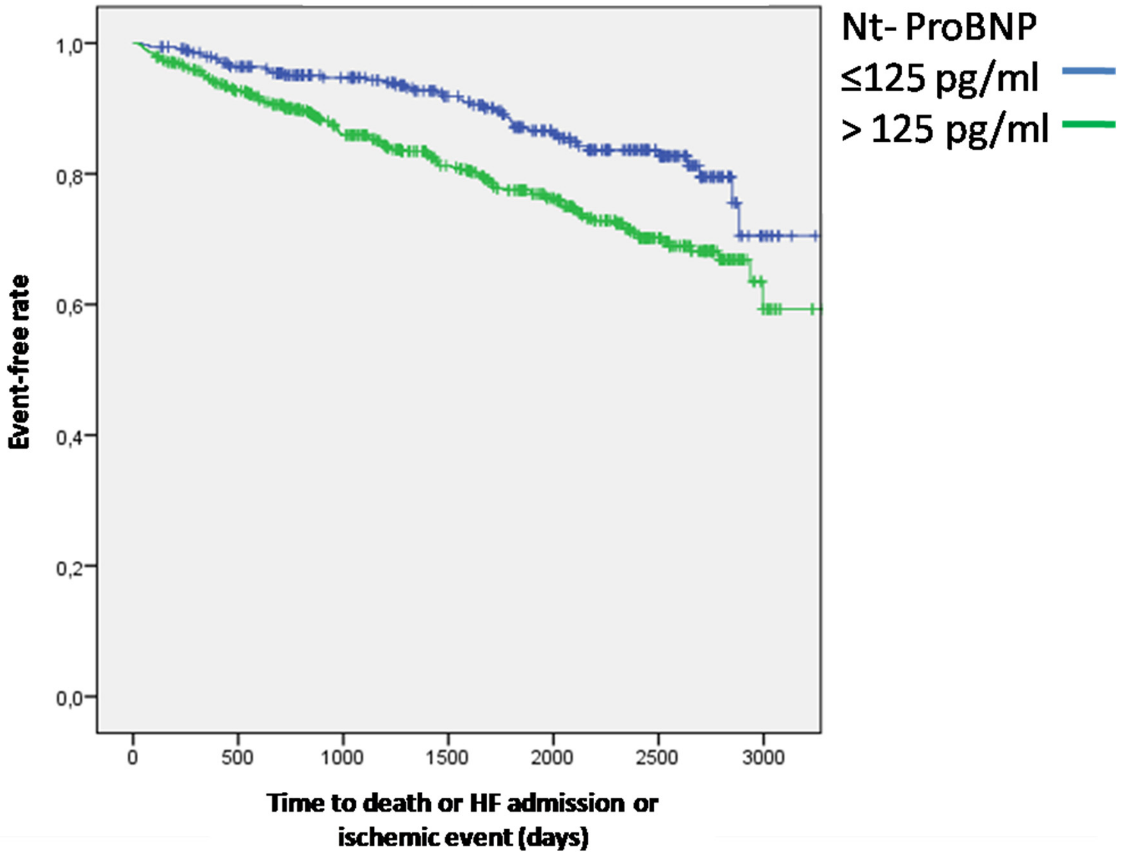

Figure S1. Kapplan-Meier curve showing time to first episode of death, HF admission or ischemic event according to NT-ProBNP levels.

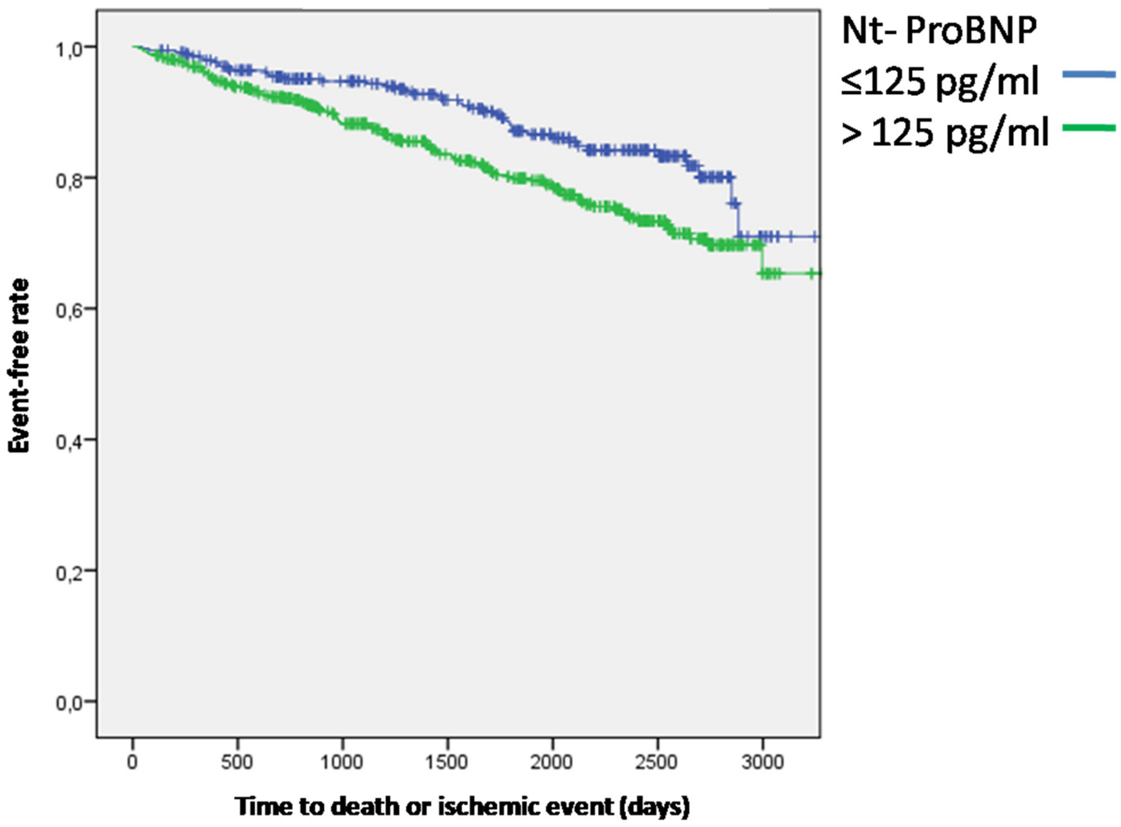

Figure S2. Kapplan-Meier curve showing time to first episode of death or ischemic event according to NT-ProBNP levels.

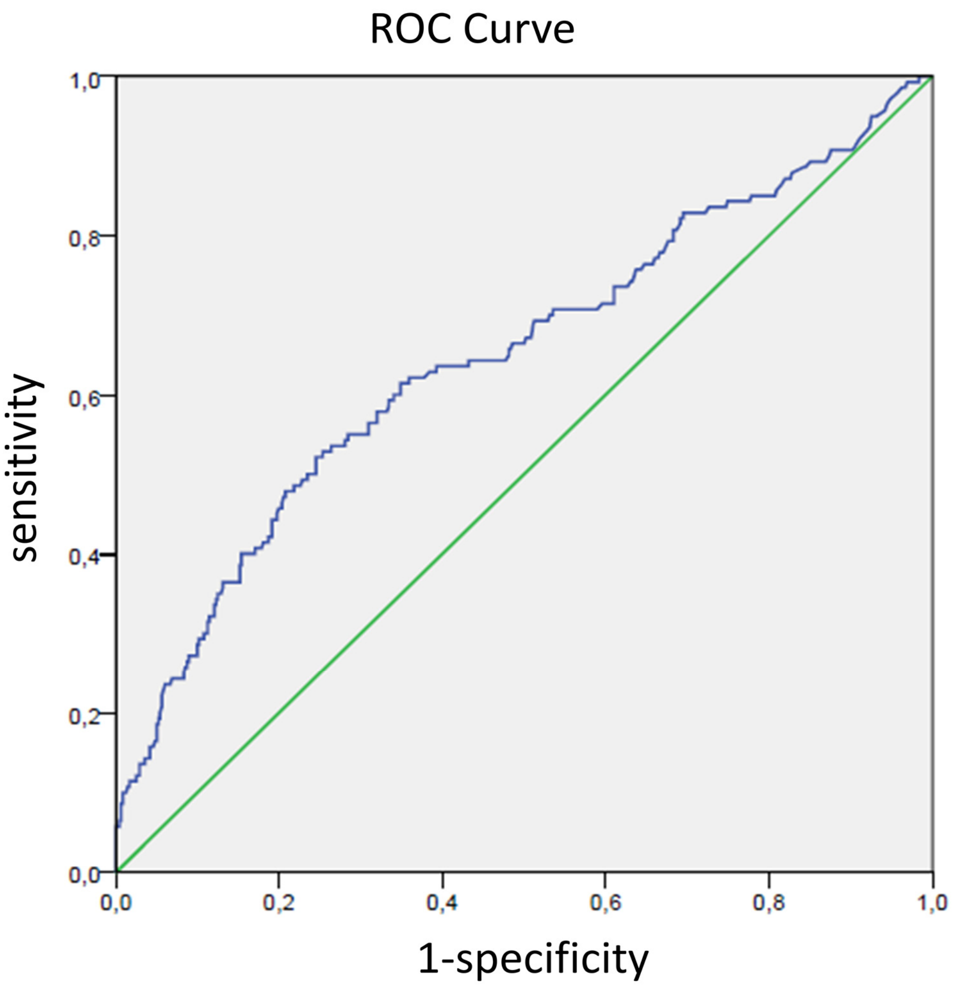

Figure S3. ROC Curve for NT-proBNP in the group of NT-proBNP > 125 pg/ml.

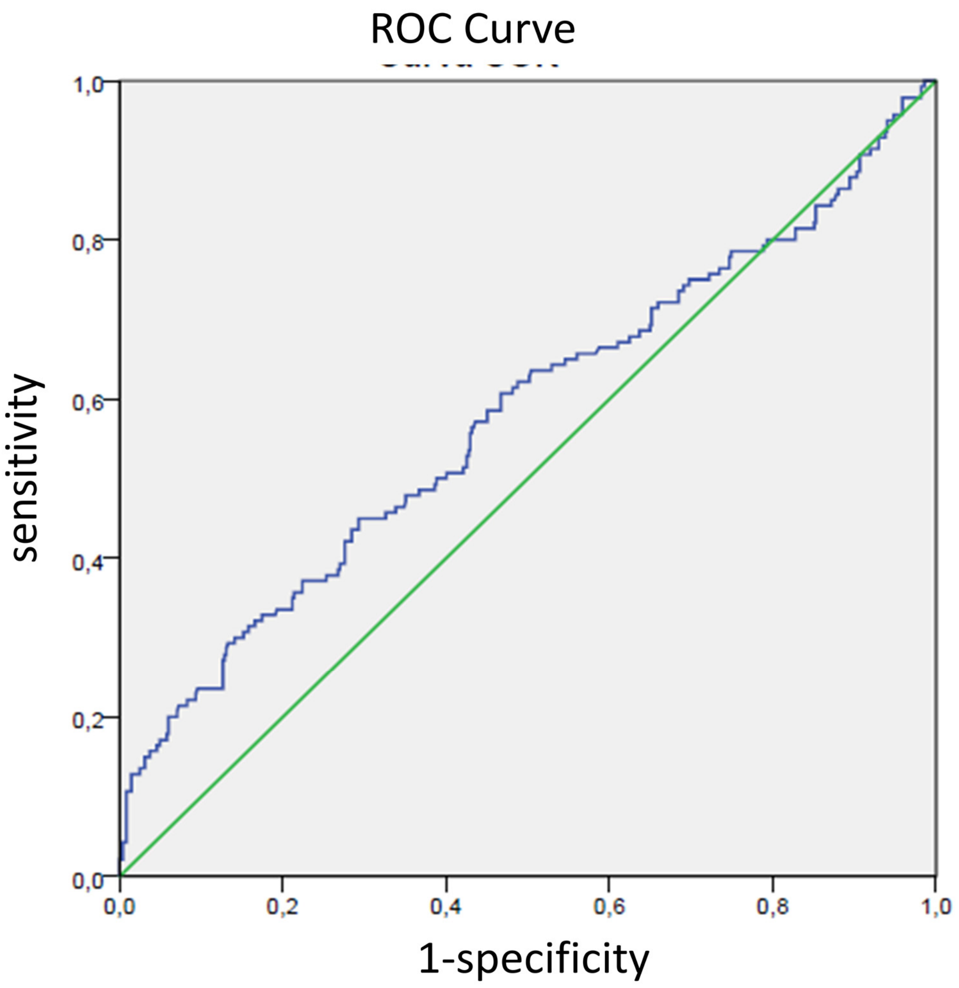

Figure S4. ROC Curve for PTH in the group of NT-proBNP > 125 pg/ml.

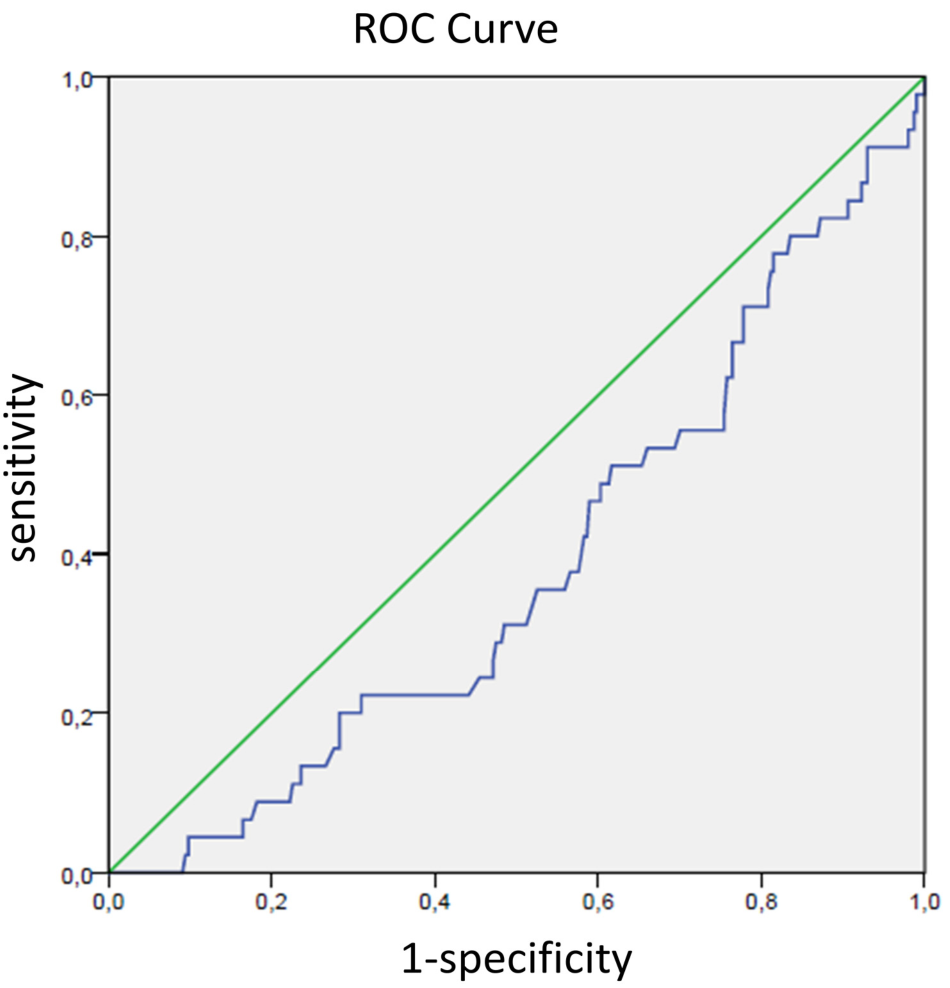

Figure S5. ROC Curve for calcidiol the group of NT-proBNP < 125 pg/ml.
